# Fingolimod-associated macular edema controlled with nepafenac non-steroidal anti-inflammatory opthalmologic applications

**DOI:** 10.1186/s12948-020-00119-4

**Published:** 2020-03-12

**Authors:** Rachel Husmann, John B. Davies, Malik Ghannam, Brent Berry, Praful Kelkar

**Affiliations:** 1 University of Minnesota, Minneapolis, MN USA; 2 VitreoRetinal Surgery, PA, 7760 France Ave S, Suite 310, Minneapolis, MN USA; 3 Department of Neurology, University of Minnesota, MMC 295, 12-181 Phillips Wangensteen Building, 516 Delaware St. SE, Minneapolis, MN 55455 USA; 4 Minneapolis Clinic of Neurology, 4225 Golden Valley Road, Golden Valley, MN 55422 USA

**Keywords:** Multiple sclerosis, Fingolimod, Retina, Macular edema, Nonsteroidal anti-inflammatory eye drops

## Abstract

**Background:**

Fingolimod, an immunomodulatory agent, is used for the treatment of relapsing–remitting multiple sclerosis (RRMS). Fingolimod-associated macular edema (FAME) is a known complication with an incidence of 0.4%. The current recommendation for treatment of FAME is cessation of fingolimod. There are few case reports with management of FAME with steroid eye drops.

**Case presentation:**

A 38-year-old Caucasian female patient with history of relapsing–remitting multiple sclerosis (RRMS) and treated with fingolimod developed Fingolimod-associated macular edema (FAME). Nevertheless, FAME was successfully treated with nonsteroidal anti-inflammatory eye drops without discontinuation of fingolimod.

**Conclusion:**

FAME may be managed with non-steroidal eye drops without discontinuation of fingolimod in appropriate patient monitored with close follow up.

## Background

Fingolimod, an immunomodulatory agent, is used for the treatment of relapsing–remitting multiple sclerosis (RRMS). Fingolimod-associated macular edema (FAME) is a known complication with an incidence of 0.4%. The current recommendation for treatment of FAME is cessation of fingolimod, which typically reverses its effects [1]. Nepafenac (brand NEVANAC) ophthalmic suspension is a nonsteroidal, anti-inflammatory prodrug indicated for the treatment of pain and inflammation associated with cataract surgery. It is dosed as one drop of NEVANAC ophthalmic suspension should be applied to the affected eye three-times-daily beginning 1 day usually and is usually prescribed in the setting of cataract surgery (for 2 weeks). The strength of formulation is in the sterile ophthalmic suspension: 0.1% 3 mL in a 4 mL bottle. Contraindications include hypersensitivity to any of the ingredients in the formula or to other non-steroidal anti-inflammatory drugs (NSAIDS). Other theoretically risk is similar to NSAIDs class including increased bleeding time due to interference with thrombocyte aggregation, delayed healing, corneal effects including keratitis. The most common adverse reactions (5 to 10%) are capsular opacity, decreased visual acuity, foreign body sensation, increased intraocular pressure, and sticky sensation. This case describes a 38-year old female with FAME successfully controlled with nonsteroidal anti-inflammatory drops without discontinuation of fingolimod. In the United States the use of nepafenac would be an off-label use of the medication in the purpose of treating FAME.

## Case presentation

A 38-year-old Caucasian female with history of RRMS initiated fingolimod (0.5 mg QD) after experiencing significant disease progression with glatiramer acetate. Her baseline ophthalmologic examination was normal.

Six months after commencing fingolimod, she developed decreased vision in the right eye and was referred to a retinal specialist for consultation. At the time of presentation, her best corrected visual acuity (BCVA) measured 20/25 in OD (right eye) and 20/20 in OS (left eye). Dilated exam showed mild macular edema (ME) and exudates in the right retina. The left retina was normal. Ocular coherence tomography (OCT) revealed moderate ME in the right macula and fluorescein angiography demonstrated corresponding leakage of dye. The left macula was anatomically and angiographically normal.

**Fig. 1:**
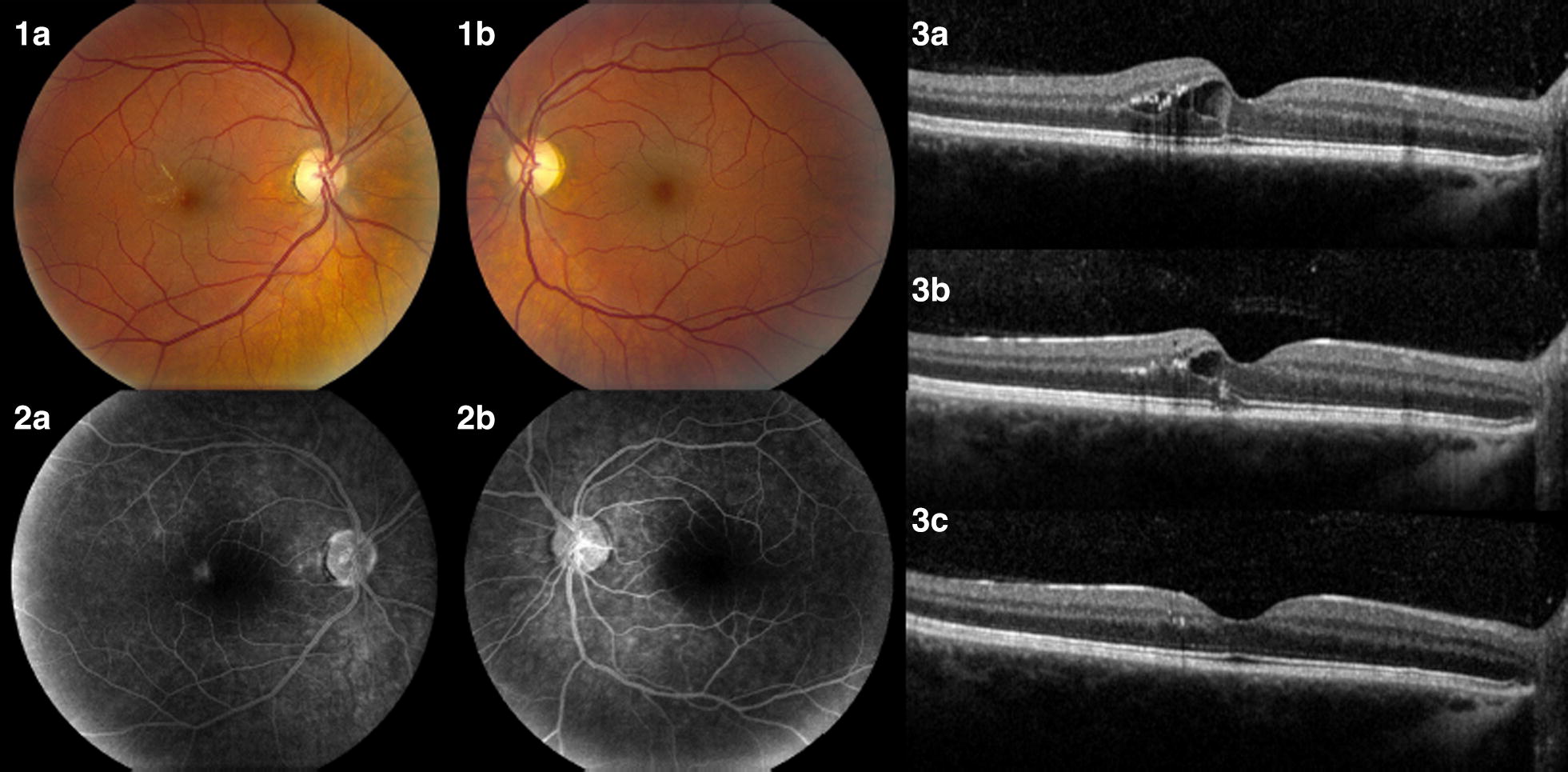
**1** Fundus at diagnosis of macular edema. The right eye has moderate ME with intraretinal exudates temporal to the fovea (**1a**). The left eye is normal (**1b**). **2** Fluorescein angiogram at presentation. The right eye has moderate leakage of fluorescein dye temporal to the fovea (**2a**). The left eye is normal (**2b**). **3** OCT of the right eye. The right eye at presentation. There is moderate cystoid ME and intraretinal exudates (**3a**). The right eye 1 month after initiating nepafenac treatment. There is a decrease in ME (**3b**). The right eye 2 years after initiating nepafenac treatment. The ME has essentially resolved with restoration of the normal foveal contour. There are minimal remaining intraretinal exudates (**3c**)

Management options for ME were discussed with the patient, including cessation of fingolimod versus starting a trial of nepafenac, a nonsteroidal anti-inflammatory drop. Since her multiple sclerosis was well-controlled with fingolimod, she was reluctant to discontinue this medication. Therefore, it was decided to continue fingolimod and start nepafenac ophthalmic drops (0.1% suspension three applications daily and one drop per application) in the right eye. The patient returned for follow-up 1 month later. Her BCVA improved to 20/20 in OD. Ophthalmologic examination and OCT showed that the ME had improved. The ME decreased over subsequent months and has resolved with ongoing nepafenac therapy. Additionally, her multiple sclerosis remains well-controlled on fingolimod with no relapses, disability, or change in MRI findings for 3 years. Look at Fig. 1.

## Discussion

Fingolimod was the first oral agent approved by the Food and Drug Administration (FDA) for the treatment of RRMS. Active metabolites of fingolimod bind to the sphinogosine-1-phosphate (S1P) receptor on lymphocytes resulting in internalization and degradation of the receptor. This action prevents lymphocytes from leaving secondary lymphoid organs, thus reducing the number of circulating lymphocytes available to participate in central nervous system autoimmune demyelination. In addition to modulating lymphocytes, the S1P receptor is responsible for regulating vascular permeability via interaction with the cytoskeleton and intracellular junctions. Disruption of the endothelial barrier in the retina may be implicated in the development of ME with use of fingolimod [1].

Data from clinical trials compiled by the FDA demonstrate the incidence of FAME to be 0.4% at the 0.5 mg dose with most cases occurring within 3–4 months of initiating treatment. The FDA recommends baseline ophthalmologic examination with repeated studies at 3–4 months. Should ME develop, resolution typically occurs after cessation of fingolimod [1].

For patients who develop fingolimod-associated ME, resolution typically occurs within 6 months following cessation of fingolimod, with 84% of patients in the pooled safety cohort having complete resolution [1, 2]. FAME was first realized not in ophthalmology or neurology patient populations but in renal transplant recipients who were treated with fingolimod as an immunosuppression agent during trials. Specifically, fingolimod at 2.5 mg/day or 5.0 mg/day, caused macular edema in 1.3% and 2.2% of patients, respectively [3]. Two trials, the TRANSFORMS and FREEDOMS trials subsequently utilized routine ophthalmic evaluation for fingolimod associated macular edema thereafter. The importance of these two studies with regard to FAME was that they found 0.2% incidence at a then lower FDA approval dosage of 0.5 mg/day. There are extensions of these trials ongoing on ophthalmic monitoring continues in both trials. Fingolimod discontinuation resulted in resolution of FAME and thus the studies mandated that should FAME develop the drug was to be discontinued. This has been incorporated into many clinical practices but leaves a problem for patients with severe demyelinating disease who have suboptimal response to other treatments. In these two trials, topical NSAIDs were occasionally prescribed by neurologists/ophthalmologists. There is minimal high level evidence to study this given that the incidence of FAME is low [4].

Possible treatment options for ME include nonsteroidal anti-inflammatory drugs, corticosteroids, vascular endothelial growth factor antagonists, laser photocoagulation, and vitreoretinal surgery. However, only a few cases report successful treatment of ME in the setting of continued fingolimod use. Such cases describe use of topical or injected corticosteroids, which are associated with serious adverse effects, such as delayed healing, infection, elevated intraocular pressure, and cataracts [5–8]. In one case fingolimod was continued while FAME was persistent and not specifically treated [9].

Nepefanac topical application may result in the following adverse reactions: hypertension (≤ 4%), headache (≤ 4%), Nausea (≤ 4%), vomiting (≤ 4%), Decreased visual acuity (≤ 10%), increased intraocular pressure (≤ 10%), conjunctival edema (≤ 5%), corneal edema (≤ 5%), eye pain (≤ 5%), eye pruritus (≤ 5%), lacrimation (≤ 5%), ocular hyperemia (≤ 5%), photophobia (≤ 5%), vitreous detachment (≤ 5%), xerophthalmia (≤ 5%), Sinusitis (≤ 4%). Nepafenac carries the following contra-indications which should be discussed with the patient prior to administration: hypersensitivity should the patient have NSAID allergy documented [10, 11].

This report describes a case of FAME successfully treated with nonsteroidal anti-inflammatory drops without discontinuation of fingolimod. The patient’s ME resolved with ongoing treatment without evidence of side effects. This provides class IV evidence for safe management of FAME with nonsteroidal anti-inflammatory eye drops without discontinuation of fingolimod.

## Conclusion

FAME may be managed with non-steroidal eye drops without discontinuation of fingolimod in appropriate patient monitored with close follow up.

## Data Availability

All the data supporting our findings is contained within manuscript.

## Abbreviations

FDA: Food and Drug Administration
S1P: Sphinogosine-1-phosphate
RRMS: Relapsing-remitting multiple sclerosis
FAME: Fingolimod-associated macular edema
BCVA: Best corrected visual acuity
ME: Macular edema
OCT: Ocular coherence tomography
OD: Oculus dexter
OS: Oculus sinister
